# Intelligent Patient Management in Viral Diseases: An Integrated Regression Model and Multi-Criteria Decision-Making Approach to Convalescent Plasma Transfusion

**DOI:** 10.3390/healthcare13243199

**Published:** 2025-12-06

**Authors:** Thura J. Mohammed, Ahmed S. Albahri, Alhamzah Alnoor, Khai Wah Khaw, Xin Ying Chew, Shiuh Tong Lim

**Affiliations:** 1 School of Computer Sciences, Universiti Sains Malaysia, Minden 11800, Pulau Pinang, Malaysia; thurajamal@student.usm.my; 2 Technical College, Imam Ja’afar Al-Sadiq University, Baghdad 10001, Iraq; ahmed.bahri1978@gmail.com; 3 College of Business Administration, A’Sharqiyah University, Ibra 400, Oman; alhamzah.malik@stu.edu.iq; 4 School of Management, Universiti Sains Malaysia, Minden 11800, Pulau Pinang, Malaysia; shiuhtong1997@gmail.com

**Keywords:** convalescent plasma transfusion, intelligent patient management, regression model, telemedicine, trustworthy AI, viral diseases

## Abstract

**Background/Objectives:** Viral diseases remain a major threat to global public health, particularly during outbreaks when limited therapeutic resources must be rapidly and fairly distributed to large populations. Although Convalescent Plasma (CP) transfusion has shown clinical promise, existing allocation frameworks treat patient prioritization, donor selection, and validation as separate processes. This study proposes a credible, converged smart framework integrating multicriteria decision-making (MCDM) and regression-based validation within a telemedicine environment to enable transparent, data-driven CP allocation. **Methods:** The proposed framework consists of three stages: (i) Analytic Hierarchy Process (AHP) for weighting five clinically relevant biomarkers, (ii) dual prioritization of patients and donors using Order Preference by Similarity to Ideal Solution (TOPSIS) and Višekriterijumsko Kompromisno Rangiranje (VIKOR) with Group Decision-Making (GDM), and (iii) regression-based model selection to identify the most robust prioritization model. An external dataset of 80 patients and 80 donors was used for independent validation. **Results:** The external GDM AHP-VIKOR prediction model demonstrated strong predictive performance and internal consistency, with R^2^ = 0.971, MSE = 0.0010, RMSE = 0.032, and MAE = 0.025. Correlation analysis confirmed biomarker behavior consistency and stability in ranking, thereby reinforcing the reliability of the prioritization outcomes. **Conclusions:** The proposed patient–donor matching framework is accurate, interpretable, and timely. This work presents an initial step toward realizing safe AI-enabled transfusion systems within telemedicine, supporting transparent and equitable CP allocation in future outbreak settings.

## 1. Introduction

This introduction is organized into three subsections. Section 1.1 highlights the relevance of this study. Section 1.2 formulates the core problem and outlines key challenges. Section 1.3 presents gaps in the literature and states the objectives of this work.

### 1.1. Motivation

Rapidly evolving viral diseases pose a critical challenge to healthcare systems, where scarce therapeutic resources must be allocated equitably, effectively, and under time pressure [1]. Those diseases, such as influenza, the common cold, HIV/AIDS, Ebola, Zika, and, more contemporarily, COVID-19, not only affect individuals with varying severity levels but also destabilize societies on a massive scale [2,3]. CP is one such resource whose success depends heavily on matching the right donor to the right patient at the right time [4,5]. In parallel, telemedicine is currently a prevailing global trend aimed at providing healthcare services as close to patients as possible [6]. This modality can play a significant role in the initial stages, such as donor recruitment for CP therapy. Healthcare professionals can virtually screen recovered patients to evaluate their suitability for CP donation, assessing their recovery timeline and overall health status [7,8]. Therefore, optimally matched CPs should be administered to the most critically ill patients with viral diseases, with telemedicine utilized to determine the most impacted measures for all blood groups [9]. Therefore, the promise of AI is a structured, explainable prioritization, integrating clinical insight with data-driven reasoning to support real-time, trustworthy decisions under uncertainty.

### 1.2. Problem Statement and Challenges

While AI has made inroads into clinical fields, CP allocation is still piecemeal in nature and very rarely, if ever, validated in practice [10]. Current systems focus on patient prioritization, donor evaluation or matching logistics, with no integrated, trusted framework for comprehensive decision support [11,12]. When CP choices may change the survival of a patient, the lack of integration results in nontransparent or non-integrated allocation, which is particularly confounding [13]. However, AI models in telemedicine also suffer from the absence of quantifiable measures of trustworthiness, a drop in performance and an inability to generalize, particularly when they are trained and tested on the same data as well as between clinical contexts [14]. Without explicit transparency, reliability metrics, or resilience to variability, clinicians remain wary of deploying AI in life-critical settings [15]. Together, these dual gaps threaten the translation of intelligent decision support from concept to safe, scalable practice.

### 1.3. Research Gap and Objectives

While artificial intelligence has been extensively investigated for healthcare decision support, current strategies regarding CP management are somewhat isolated and not well tested [16]. Prior works have presented prioritization models or donor selection but not a unified empirically validated approach that satisfies transparency, dependability and adaptability in the context of real-time telemedicine [17,18]. The majority of systems being developed lack instruments to assess confidence in the consistency of decisions and methods within a dynamic clinical environment, introducing insecurity in automated decision recommendations [19]. To fill these gaps, in this study, a trustworthy integrated intelligent framework is proposed that combines patient–donor prioritization and validation into a single architecture. The model combines data-driven analysis and expert-informed judgment to determine the optimal CP for critical patients given ethical and operational considerations [20]. Through the integration of verification and trust assessment processes within the decision workflow, the framework offers strengthened transparency, consistency and clinical confidence [21,22]. The main purpose of this study is thus to establish and assess a robust and transparent CP distribution mechanism capable of efficiently functioning in telemedicine settings. The framework is intended to be useful in developing strategies for backed-by-data and trustworthy intelligent health information during the next public health crisis, as well as in enabling more trustful real-time matching between patients and donors, as well as improving the credibility of AI-assisted healthcare decisions.

## 2. Related Works

### 2.1. Decision-Support Frameworks for CP and Viral Disease Management

AI-based medical decision support research has been expedited due to the COVID-19 pandemic, resulting in a variety of frameworks for resource allocation, triage and treatment prioritization [23]. A number of other works have examined multicriteria or data-driven systems to rank patients-donors, proving that AI could lead to a decrease in human factors and swift response times in disaster situations [24]. For example, early CP systems used expert-defined biomarker weights and automatic scores to select critically ill patients and ideal donors with applications in hospital networks and telemedicine since their allocation accuracy has increased [25]. However, these systems are limited in that they are designed to handle only one of the decision stages (patient prioritization, donor selection or CP matching) and do not apply the three processes together as a coherent pipeline [26]. Parallel research in broader clinical AI confirms this gap: most systems achieve strong internal accuracy but lack cross-validation or empirical reliability testing, particularly when deployed remotely or across heterogeneous datasets [27]. Moreover, transfusion safety studies emphasize that ABO CP compatibility rules must be embedded in computational models rather than applied post hoc, as incompatibilities can compromise patient safety [28]. Collectively, the literature underscores both the feasibility and the incompleteness of current CP decision frameworks, highlighting the need for end-to-end, data-validated, and operationally consistent solutions suitable for telemedicine workflows.

### 2.2. Trustworthy and Transparent Artificial Intelligence in Telemedicine

Recent studies have noted that, in addition to technical optimization, developing trustworthy, explainable and robust AI-based solutions is among the requirements for successful AI adoption in healthcare [29]. The WHO and FUTURE-AI guidelines specify that medical AI should perform well with a distributional shift (robustness), have human-in-the-loop oversight for decision-making, and interpret why each recommendation was made [30]. Systematic reviews of trusted and explainable AI in healthcare continue to find deficiencies in transparency and bias analysis, especially outside of clinical contexts or with data scarcity [31]. Telemedicine, by extending care to the community, exacerbates these concerns: clinicians now make decisions on the basis of algorithmic output without seeing the patient; thus, credible reliability and ethical safeguards are crucial [14]. Works on AI-based triage, telemonitoring and decision fusion frameworks demonstrate high diagnostic performance, but they tend to overlook a rigorous manner to quantify or validate model trust [24]. Thus, the literature points in a clear direction regarding the essence of AI systems, which embed trust metrics that can be measured, such as consistency, robustness and cross-validation, in their operational part.

## 3. Framework Methodology

**Figure 1:**
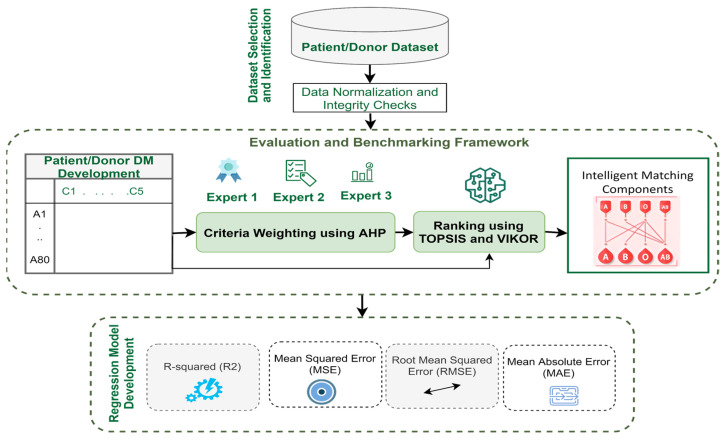
Research methodology phases.

The design of the model is based on a three-stage process. The initial phase, dataset selection and identification, is an essential stage for selecting an appropriate viral disease dataset for model construction. The second phase is a trust-based decision-making model development phase that aims at building a trusted and intelligent framework adapted to CP transfusion. The last phase of regression model selection is the stage of constructing a proposed predictive model that can efficiently search for the best-fitted model. This framework aims to rank viral disease patients and donors according to their order. The steps of the research methodology process are depicted in Figure 1.

The framework will interface with telemedicine platforms via a RESTful API supporting JSON format and OAuth2 authentication for secure data exchange. Alerts will notify clinicians if biomarker values exceed predefined thresholds, ensuring timely intervention. Fail-safe mechanisms will allow clinicians to access manual overrides and redundant backups to minimize disruptions.

### 3.1. Dataset Selection and Identification

The dataset employed in this study was sourced from a previously published and peer-reviewed clinical investigation with fully validated biomarker records [14]. This dataset originated from a single-center clinical study published in a high-impact peer-reviewed journal. The patient and donor data were collected within the original study’s defined time window, covering the clinical assessment period of viral disease cases during the 2021 outbreak. All clinical records were fully anonymized prior to publication, and the dataset was made available in compliance with institutional ethical regulations. As the dataset was released with only complete biomarker profiles, no missing values were present, and therefore no imputation or missingness-handling procedures were required.

Following a comprehensive analysis derived from a literature review, this study found that this dataset was suitably comprehensive, as it encapsulates all the necessary criteria for a CP transfusion framework. This dataset distinctly classifies 80 patients and 80 donors on the basis of five biomarker criteria (PAO2/FIO2, CRP, IL-6, albumin, and IgM) and spans four blood types (A, B, AB, and O) [32,33]. Specifically, in the context of viral diseases, it has emerged as a potentially valuable resource for future research because it has been both validated and verified by medical experts. In accordance with the structure of the original dataset, the index date was defined as the baseline clinical assessment at which all five biomarkers were recorded. The inclusion criteria therefore consisted of (i) confirmed viral disease diagnosis, (ii) complete measurement of all biomarker values, and (iii) verified ABO blood typing. Individuals with missing biomarkers, unverified clinical data, or incomplete ABO records were excluded. As the dataset contains a single baseline biomarker profile per patient and per donor, no repeated or longitudinal measurements were present [13,34].

The dataset was managed via Microsoft Excel, the data ranges were normalized, and the data matrices were structured. All biomarker values were confirmed to be within their medically accepted ranges, ensuring data integrity and reliability. An extract showing the first patient/donor for each blood type is shown in Table 1.

For biomarkers, the PAO2/FIO2 ratio (the arterial oxygen tension/fraction of inspired oxygen) should be between 100–300 [35]. C-reactive protein (CRP) is a key member of the pentraxin family and functions as an index of inflammation, with a reference range of 8–250 [36]. IL-6 is a product of T cells and activated macrophages during the acute-phase response to trauma or injury that can be used to predict inflammation or infection (aortic rupture), with cutoff values between 6 and 300 [37]. Albumin, an important binding and transport protein, contributes to the maintenance of osmotic pressure in blood [38], within which reference values are generally set between 5 and 55. Finally, the enzyme-linked immunosorbent assay (ELISA) test, which detects immunoglobulin M (IgM) and immunoglobulin G (IgG) antibodies against Haemophilus influenzae antigens, should yield values between 100 and 800 [39,40].

To reinforce the clinical rationale behind the selected biomarker set, this study further clarify that each criterion reflects a clinically validated dimension of viral disease severity. The PAO_2_/FiO_2_ ratio is a primary indicator of respiratory failure and is commonly used for triage in acute respiratory distress. CRP and IL-6 reflect the systemic inflammatory response and cytokine activation characteristic of viral pneumonia progression and cytokine storm, respectively. Albumin indicates nutritional and immunological resilience, where hypoalbuminemia is strongly associated with higher mortality in severe viral infections. Finally, IgM represents early humoral immune activity and informs the therapeutic potential of donor plasma. Together, these biomarkers mirror standard clinical decision pathways and provide a medically interpretable basis for triage within CP transfusion workflows.

### 3.2. Trust Decision-Making Model Development

This phase includes a three-stage development process, as illustrated in Figure 1. This process can be achieved via a real-time telemedicine application workflow in the same process. All three stages in this phase were implemented via a combination of Python 3.9 and Microsoft Excel 2016. Custom Python scripts were written via the NumPy and SciPy libraries to automate matrix normalization, consistency checks, and weight computations for the AHP. The decision matrix (DM) for patients-donors was structured in CSV format and processed programmatically. Where needed, intermediate analyses, such as eigenvector computation and consistency ratio calculations, were verified in Excel to crosscheck the outputs. This ensured full traceability and replicability across software environments.

#### 3.2.1. Stage 1: DMs for Patients-Donors Adoption

The DMs in this study are created by considering the overlap between the biomarker criteria and the patient-donor list. However, the management of patients-donors suffering from viral illness is a special challenge and will require different models of decision-making to allow for prioritization of care [41,42]. The first aim of this holistic strategy is to greatly reduce the complexity that the study addresses in managing the problem. DMs were created by mapping patient and donor biomarker values to structured NumPy arrays. The matrices were verified for completeness and normalized before use in the MCDM models. This was undertaken to guarantee that the input data for the AHP, TOPSIS and VIKOR models were formatted in a similar manner among the 160 samples. Table 2 shows both DMs.

#### 3.2.2. Stage 2: Integrated AHP-TOPSIS and AHP-VIKOR

**Figure 2:**
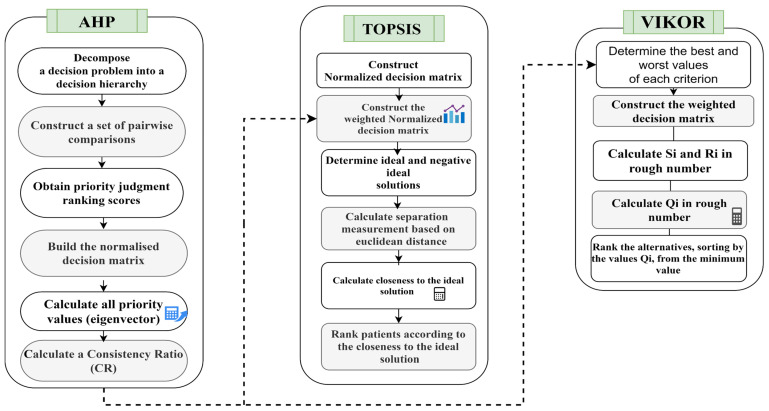
Integrated AHP–TOPSIS and AHP–VIKOR models for prioritization.

This study proposes MCDM approaches to solve decision problems on the basis of multiple criteria. MCDM can help promote a fair, transparent and rational priority setting process that is widely seen as one of the most difficult processes from multiple medical rationing perspectives [43]. The study follows a few steps for objectively assigning the values of weights to the biomarker criterion to ensure free-of-bias methodology. The research uses the AHP method with the TOPSIS and VIKOR methods to prioritize patient donors. The combined steps are shown in Figure 2.

**A. AHP for Setting Weights for Biomarker Criteria**

This section provides further details regarding the methodology employed for assigning weights and proposes a specific approach for assigning subjective weights to the criteria related to viral diseases in patients and donors, utilizing the AHP method. Furthermore, this study aims to investigate the relevant criteria for conducting a comprehensive analysis pertaining to both recipients and contributors. The AHP technique is outlined in the following steps [44]:

STEP 1: Decomposition of a Decision Problem into a Decision Hierarchy

**Figure 3:**
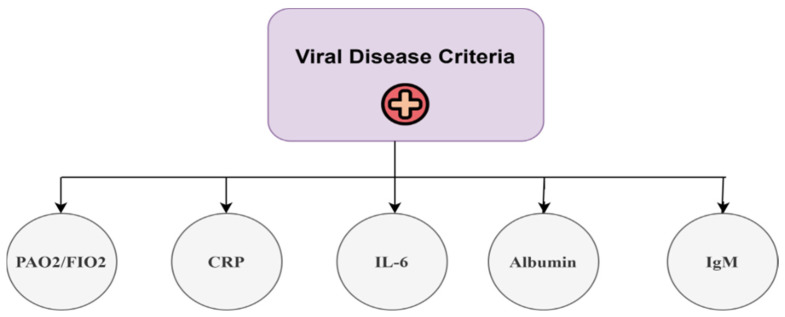
Hierarchy of the AHP for the viral disease criteria.

The issue pertaining to hierarchical modeling entails the development of a decision objective that necessitates structuring in accordance with the criteria stated in the AHP [44]. Figure 3 illustrates the hierarchical arrangement of the criteria utilized in the AHP pairwise comparison for the assessment of criterion weights related to biomarkers. The apex of the hierarchical framework represents the highest goal, which is achieved by meeting the five set requirements. Conducting pairwise comparisons among all criteria is deemed essential [45].

Step 2: Construction of the pairwise comparison matrix

**Figure 4:**
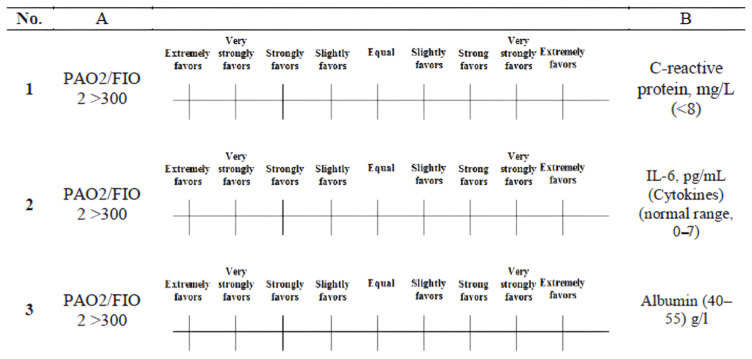
Representative example of the pairwise comparison judgment form.

The AHP can build a pairwise comparison matrix to establish a decision, as shown in Equation (1):

(1) $$A=\left(\begin{array}{ccccc} x_{11} & x_{12} & \ldots & \ldots & x_{1n}\\ x_{21} & x_{22} & \ldots & \ldots & x_{2n}\\ \vdots & \vdots & \vdots & \ddots & \vdots \\ x_{n1} & x_{n2} & \ldots & \ldots & x_{nn} \end{array}\right)\;\mathrm{where},\;\left\{\begin{array}{c} x_{ii}=1\\ x_{ji}=\frac{1}{x_{ij}} \end{array}\right.$$

where the elements X*_ij_* are obtained from Figure 3. The relative value of each criterion is assessed via a number scale ranging from 1 to 9 [46]. Table 3 presents the relative scales ranging from 1–9, which were employed to depict the judgments of each expert for each comparison [47]. Professionals must evaluate these judgments in a critical manner, drawing upon their expertise and knowledge [48]. The pairwise comparison data were collected via structured questionnaires distributed via Google Forms to three domain experts in pulmonology and infectious diseases. A representative sample of the expert judgment form used to obtain the pairwise comparison values is shown in Figure 4, illustrating the application of the standard 1–9 semantic scale. The responses were exported and converted into 5 × 5 comparison matrices for each expert.

STEP 3: Obtaining priority judgment ranking scores

A questionnaire for pairwise comparison was developed and disseminated to a geographically diverse convenience sample of experts [49]. The three domain experts contributing to the AHP stage were infectious disease specialists with 10–22 years of clinical experience. The three experts were requested to demonstrate their assessments and the relative significance for all criteria through the utilization of the nine scales for comparison. The formula for determining the number of necessary pairwise comparisons is n × (n − 1)/2, where n represents the total number of criteria utilized in the evaluation process [50]. In cases of disagreement within pairwise comparisons, experts discussed discrepancies until a consensus matrix was reached with a Consistency Ratio (CR) < 0.1, thus ensuring methodological rigor and minimizing subjectivity. At this juncture, the AHP derives the relative weights of significance for all criteria related to viral diseases through pairwise comparisons on the basis of user preferences and judgments provided by the decision-making team [51].

STEP4: Construction of the normalized DM

The normalization process involves dividing each element in matrix A by the sum of the elements in the corresponding column [52]. This results in the creation of a normalized pairwise comparison matrix, denoted as Anorm. The matrix A (1) is normalized to obtain the matrix Anorm. The elements of A (xij) are determined via Equation (2).

(2) $$a_{ij}=\frac{x_{ij}}{\sum_{i=1}^{n}xij}$$

STEP5: Calculation of all priority values (eigenvectors)

The AHP pairwise comparison method employs mathematical calculations to convert subjective judgments into weights for all criteria. Once the responses for the pairwise comparisons are collected, a reciprocal matrix is constructed on the basis of these comparisons. The weights of decision factor i can then be determined via Equation (3):

(3) $$w_{i}=\frac{\sum_{j=1}^{n}a_{ij}}{n}and\sum_{j=1}^{n}w_{i}=1$$

where n is the number of compared elements. The AHP measurement steps must be designed to obtain weights on the basis of the evaluator’s preference.

STEP6: Calculation of the CR

The CR, which expresses the internal consistency of judgments, is determined. The following concepts are specified to establish a quantitative measure of the degree of inconsistency within a pairwise comparison matrix [43]. The consistency index (CI) is calculated via Equations (4) and (5):

(4) $$CI=\frac{\lambda \;\max-n}{n-1}$$

(5) $$RI=\frac{1.98(n-1)}{n}.CI$$

The measure of inconsistency, known as the CI, quantifies the extent of discrepancy. The RI is used to quantify the level of inconsistency present in a pairwise comparison matrix. The variable CR is denoted and mathematically expressed in Equation (6):

(6) $$CR=\;\frac{CI}{RI}$$

The CR is a numerical indicator used to quantify the extent of inconsistency present in a pairwise comparison matrix. The pairwise comparison matrix should have a CR that does not exceed 10% or 0.1. If the obtained weights meet this criterion, they are considered acceptable [53]. However, if the obtained weights do not meet this criterion, they should be disregarded, and decision-makers should be requested to complete the designed questionnaires again until an acceptable CR ratio is achieved.

The MCDM weights in this framework represent expert-driven prioritization based on clinical severity markers (e.g., PAO_2_/FiO_2_, CRP, IL-6, albumin, and IgM). These biomarkers guide patient prioritization for CP transfusion; however, the system is intended as an operational triage tool rather than a predictor of patient outcomes. The prioritization scores are not directly linked to clinical outcomes, such as recovery rates, which depend on a range of clinical factors outside the scope of this decision-support model.

**B. TOPSIS for the Prioritization of Patients-Donors**

During this phase, the TOPSIS technique is employed as the initial approach for prioritizing patients or donors. This is done by utilizing the weighted criteria derived from the AHP method. The objective is to address a significant drawback of the TOPSIS method, namely, the absence of weights provided for the evaluation criteria. The procedural framework of the TOPSIS method [54,55] is as follows:

STEP 1: Constructing the normalized DM

The transformation of attribute dimensions into nondimensional attributes enables meaningful comparisons across various qualities. The matrix (xij)m*n is subsequently subjected to normalization, resulting in the matrix that employs the normalization technique outlined in Equation (7) [56].


(7) $$r_{ij}=x_{ij}/\sqrt{\sum_{i=1}^{m}\;\;x_{ij}^{2}}$$

STEP 2: Constructing the weighted normalization DM

In this procedure, the decision-maker’s set of weights, denoted wi = w1, w2, w3, …, wn, is applied to the normalized DM, as presented in Equation (8). The resulting matrix can be obtained by multiplying each column of the normalized DM by its corresponding weight wj [57]:


(8) $$\sum_{j=1}^{m}\;w_{j}=1$$

STEP 3: Determining the ideal and negative ideal solutions

In this process, two artificial alternatives, A* (the ideal alternative) and A− (the negative ideal alternative), are defined by Equations (9) and (10), respectively:

(9) $$\begin{array}{cc} A*= & \left\{\left(\left({max}_{i}\;v_{ij}|j\in J\right),\left({min}_{i}\;v_{ij}|j\in J^{-}\right)|i=1,2,\ldots ,m\right)\right\}\\ & =\left\{v_{1}^{*},v_{2}^{*},\ldots ,v_{j}^{*},\ldots v_{n}^{*}\right\} \end{array}$$

(10) $$\begin{array}{cc} A^{-}= & \left\{\left(\left({min}_{i}\;v_{ij}|j\in J\right),\left({max}_{i}\;v_{ij}|j\in J^{-}\right)|i=1,2,\ldots ,m\right)\right\}\\ & =\left\{v_{1}^{-},v_{2}^{-},\ldots ,v_{j}^{-},\ldots v_{n}^{-}\right\}, \end{array}$$

where j is the subset of, which presents the benefit attribute (i.e., offering an increasing utility with high values), and where $J^{-}$ is the complement set of j. The opposite can be added for the cost-type attribute denoted by $J^{c}$.

STEP4: Separation measurement calculation based on the Euclidean distance

In this process, the separation measurement is performed by calculating the distance between each alternative in V and the ideal vector A* and the negative ideal A− via the Euclidean distance, as illustrated in Equations (11) and (12):

(11) $$S_{i*}=\sqrt{\sum_{j=1}^{n}{\left(v_{ij}-v_{j}^{*}\right)}^{2}},i=(1,2,\cdots m)$$

(12) $$S_{i^{-}}=\sqrt{\sum_{j=1}^{n}{\left(v_{ij}-v_{j}^{-}\right)}^{2}},i=(1,2,\cdots m)$$

Upon completion of step 4, two distinct values, specifically S*i and S−i, are computed for each alternative. These values serve as indicators of the respective distances between each alternative and both the ideal and negative ideal states.

STEP 5: Closeness to the A*

In this process, the closeness of Ai to the ideal solution A* is defined, as shown in Equation (13).

(13) $$C_{f}=S_{i^{-}}/\left(S_{i^{-}}+S_{i^{-}}\right),0<C_{i*}<1,i=(1,2,\cdots m)$$

where C*i = 1 if and only if Ai = A−; similarly, C*i = 0 if and only if Ai = A−.

STEP 6: Alternative Ranking

The collection of alternative Ai can be prioritized according to the decreasing order of C*i, where a greater value indicates superior performance.

**C. VIKOR for the Prioritization of Patients-Donors**

The VIKOR technique is employed as a secondary approach to prioritize patients/donors by using the weighted criteria derived from the AHP method [58,59]. The patients/donors are assessed and categorized on the basis of biomarkers, utilizing the VIKOR method with the Q value as the basis for ranking them from the least to the most favorable health state. The Q value (order 1) that corresponds to the highest efficacy indicates the identification of individuals with a serious health condition. This technique suggests that these patients will possess the lowest Q value in comparison to others. Within the same context pertaining to the donors, the Q value at the lowest rank (80th) offers insight into the donors who possess an adequate CP, as shown by this methodology, and are expected to exhibit the highest Q value relative to their counterparts. The steps of the VIKOR method are as follows:

STEP 1: Determine the best and worst values of all criterion functions, i = 1; 2; …; n. If the ith function represents a benefit, then is the maximum and is the minimum; otherwise, the ith function represents a cost and then is the minimum and is the maximum.

STEP 2: In this process, the weights for each criterion (AHP weights) are introduced to VIKOR. A set of weights from the decision-maker is accommodated in the DM; this set is equal to 1 [60]. The resulting matrix can also be calculated as illustrated in Equation (14), which will produce a weighted matrix.

(14) $$WM=wi*(f*i-fij)/(f*i-f^{-}i)$$

This process produces a weighted matrix as follows:

$$\left[\begin{array}{cc} \begin{array}{cc} w_{1}(f*1-f11)/(f*1-f^{-}1\;) & w_{2}(f*2-f12)/(f*2-f^{-}2\;)\\ w_{1}(f*1-f21)/(f*1-f^{-}1\;) & w_{2}(f*2-f22)/(f*2-f^{-}2\;) \end{array} & \begin{array}{cc} \ldots & w_{i}(f*i-fij)/(f*i-f^{-}i\;)\\ \ldots & w_{i}(f*i-fij)/(f*i-f^{-}i\;) \end{array}\\ \begin{array}{cc} \vdots & \vdots \\ w_{1}(f*1-f31)/(f*1-f^{-}1\;) & w_{2}(f*2-f32)/(f*2-f^{-}2) \end{array} & \begin{array}{cc} \vdots & \vdots \\ \ldots & w_{i}(f*i-fij)/(f*i-f^{-}i\;) \end{array} \end{array}\right]$$

STEP 3: Compute the values Sj and Rj, j = 1, 2, 3, …, J, i = 1, 2, 3, …, n by using Equations (15) and (16).

(15) $$Sj=\sum_{i=1}^{n}wi*(f^{*}i-fij)/(f^{*}i-f^{-}i)$$

(16) $$Rj=\underset{i}{\max}\;wi*(f^{*}i-fij)/(f^{*}i-f^{-}i)$$

where wi are the weights of the criteria expressing their relative importance.

STEP 4: Compute the values of Q_j_ and $j=\left(1,2,\cdots ,J\right)$ via the following relation:

(17) $$Q_{j}=\frac{v\left(S_{j}-S*\right)}{S^{-}-S*}+\frac{\left(1-v\right)\left(R_{j}-R*\right)}{R^{-}-R*}$$

where $S^{*}=\underset{j}{\min}\;S_{j}$, $S^{-}=\underset{j}{\max}\;S_{j}$, $R^{*}=\underset{j}{\min}\;R_{j}$, $R^{-}=\underset{j}{\max}\;R_{j}$, and the variable v represents the weight assigned to the strategy of either “the majority of criteria” or “the maximum group utility” in this study. In the present research, the value of v is set at 0.5.

STEP 5: Alternative Ranking

The collection of possibilities, consisting of patients and donors, can be ranked by arranging them in ascending order on the basis of the value of Q.

In this proposed model, the GDM approach is utilized to enhance the decision-making process [22,59]. The GDM involves integrating the results of several decisions made by three experts into a singular decision by employing the arithmetic mean. This study employs the GDM approach to amalgamate the ranking outcomes derived from individual expert preferences. These rankings are then consolidated to produce a final prioritization of patients and donors. The use of aggregation techniques can potentially mitigate variability in expert outcomes, thereby enabling the formulation of a singular and unified ranking.

#### 3.2.3. Stage 3: Intelligent Matching Components

The phase describes how to create standards allowing reliable and efficient matching between patients and compatible donors according to the ABO rules for CP transfusions [61]. The final result of the proposed model is the assignment of suitable donors for each patient through the use of ranking methods such as AHP-TOPSIS and AHP-VIKOR. By the end of this stage, convincing evidence will have been provided of the safe, successful administration of sufficient compatible blood products from suitable donors to patients in need.

### 3.3. Regression Model Development

In this phase, through a regression model, the best prioritization model is selected on the basis of the results from both external and internal comparisons of the GDM TOPSIS and VIKOR approaches. Regression analysis is a well-known multivariate method that is widely used, particularly in social science research [62]. A possible solution is to parametrize the observed prioritization mechanism directly via multiple regression models. Model fitting can be accomplished via cross-validation methods [63,64,65]. In the regression analysis, the dependent variable represents the final prioritization score obtained from the MCDM models, whereas the independent variables consist of the biomarker-based decision matrix and the aggregated AHP-derived weights. This design allows the regression model to approximate the prioritization behavior of each decision configuration and quantify how reliably the MCDM pipeline transforms clinical features into ranked outputs. This flexible model can be used when there are single or multiple explanatory factors, and it can handle properly coded categorical explanatory variables as well [66,67]. The performance of the regression model can be gauged via several assessment measures, including the R-squared (R2), mean squared error (MSE), mean absolute error (MAE), and root mean squared error (RMSE) [68]. The R2 metric assesses the overall goodness of fit of a regression model. It represents the proportion of the total variability in the dependent variable Y accounted for by the model [69]. A higher R2 value signifies a smaller discrepancy between the observed data and fitted values, indicating better model fit, as presented in Equation (18).

(18) $$R^{2}=1-\frac{\sum_{i=1}^{n}\;\;{\left(y_{i}-{\overset{`}{y}}_{i}\right)}^{2}}{\sum_{i=1}^{n}\;\;{\left(y_{i}-\overset{⃐}{y}\right)}^{2}}\;\;\;\;\;$$

Moreover, the MAE measures the absolute difference between the observed and predicted values, as presented in Equation (19).

(19) $$MAE=\frac{1}{n}\sum_{i=1}^{n}\;\;\left|y_{i}-{\overset{`}{y}}_{i}\right|$$

The MSE is similar to the MAE but involves taking the square of the difference between the actual and predicted values; see Equation (20). A smaller MSE value indicates better model performance.

(20) $$MSE=\frac{1}{n}\sum_{i=1}^{n}\;\;{\left(y_{i}-{\overset{`}{y}}_{i}\right)}^{2}$$

The RMSE, as its name suggests, is the square root of the mean squared error. A smaller RMSE indicates better model accuracy if the predicted responses align closely with the actual responses (see Equation (21)).

(21) $$RMSE=\sqrt{\frac{1}{n}\sum_{i=1}^{n}\;\;{\left(y_{i}-{\overset{`}{y}}_{i}\right)}^{2}}$$

where $y_{i}$ represents the actual value, ${\overset{`}{y}}_{i}$ denotes the predicted value, $\overset{⃐}{y}$ is the mean of the observed values, and n is the number of observations.

## 4. Results and Discussion

This section provides a multilevel evaluation of the proposed framework, analyzing its performance in terms of biomarker weighting, prioritization accuracy, and intelligent matching. Additionally, this section presents the findings of the regression analysis. The evaluation of the regression model employed in this work principally involves the utilization of assessment measures, including R2, MSE, MAE, and RMSE, to examine its performance. This segment emphasizes the model’s dependability and validity by demonstrating its predicted accuracy and goodness of fit.

### 4.1. Weighting Results

Table 4 presents the AHP-derived weights assigned by the three medical experts to the five biomarkers. Across all the experts, C1 and C3 consistently obtained the highest weights, confirming their dominant clinical importance in assessing disease severity. The emphasis on oxygenation and the inflammatory response aligns with the current medical consensus that respiratory efficiency and cytokine activity are decisive indicators of CP transfusion prioritization. Minor variations among the experts were observed; one prioritized C5 due to its immunological relevance in donor evaluation, yet all agreed on the lower influence of C2 and C5. The consistency ratios (0.07, 0.09, 0.06) were less than 0.1, validating coherent and bias-free pairwise judgments. After aggregation through the GDM process, the unified weighting vector reflects a balanced consensus where C1 and C3 dominate while C2 and C4 act as supportive stability factors. The results establish a reliable foundation for the subsequent TOPSIS and VIKOR ranking procedures, ensuring that patient-donor prioritization is guided by clinically meaningful and statistically consistent criteria.

### 4.2. Ranking Results

Table 5 presents the results for the first ten patients and donors obtained through these four configurations. The comparative analysis of these rankings provides insight into the performance consistency of the models and the stability of the prioritization process across different decision-aggregation strategies. For the TOPSIS model, the external GDM yielded highly stable rankings, with P3_AB, P4_O, and P14_O emerging consistently as the top three critical patients, with respective scores of 0.7471, 0.7289, and 0.7215. These relatively high values of the closeness coefficient (C*) indicate a small distance from the ideal solution, indicating that severe clinical cases require immediate attention. On the donor side, D20_AB scored the highest (0.6801), followed by D8_A (0.6114) and D17_A (0.5868), identifying them as the most suitable CP sources with optimal biomarker profiles. A similar trend is observed under the internal GDM TOPSIS configuration, where P3_AB and P4_O maintain their dominant positions with scores of 0.7429 and 0.7258, respectively, confirming the reliability of the patient ranking across different expert aggregation settings. The marginal variation between the external and internal scores (less than 0.01 for most cases) demonstrates the model’s robustness to expert weight variability. In contrast, the VIKOR model applies a compromise-ranking approach based on utility and regret measures, thereby emphasizing the trade-off between group benefit and individual stability. In this case, lower Q values represent higher priority. The external GDM VIKOR ranking identifies P8_A (Q = 0.0580), P16_O (Q = 0.0853), and P20_A (Q = 0.1703) as the top three most critical patients, whereas D11_O (Q = 0.000) and D20_O (Q = 0.1663) occupy the leading donor positions. The same patients (P8_A and P16_O) also dominate the internal GDM VIKOR list, achieving comparable Q values of 0.002 and 0.014, respectively. This close alignment between the internal and external GDM results suggests that expert consensus exerts minimal influence on the VIKOR-derived compromise ranking, confirming that prioritization is data driven and not overly sensitive to subjective judgments.

### 4.3. Matching Components Results

The outcome of this part is derived from the importance of the four aforementioned principles that have been introduced to appropriately match a potential donor with a compatible patient. This matching process is carried out after the prioritization has been determined via the TOPSIS and VIKOR methods. Specifically, Table 6 shows the findings obtained from the AHP-TOPSIS and AHP-VIKOR approaches.

In the context of external GDM AHP-TOPSIS, the process of matching patients and donors can be facilitated through the utilization of an inverted relationship between them. The patient identified as P3_AB, who achieved an order of 1 with a score of 0.74719, was regarded as the most severe case among all patients. The optimal donor for this patient is D12_B, the last donor in the sequence, who attained an order of 80 with a score of 0.09808. In the context of external GDM AHP-VIKOR, the matching process between patients and donors can be facilitated through the utilization of an inverse relationship between them. As an illustration, the patient identified as P8_A, who received an order of 1 with a score of 0.05806, is regarded as the most severe case among all patients. The optimal donor for this patient is the donor designated D11_A, who received order 80 with a score of 0.95325.

To illustrate local explainability, we provide case study examples of how specific patients and donors are prioritized based on their biomarker profiles. For example, patient P1, who has low PAO_2_/FiO_2_ and elevated CRP, is ranked higher for CP allocation, while donor D5 with high IgM is preferred for matching. These examples highlight the local impact of biomarker values on decision-making and ensure transparency for clinicians.

### 4.4. Regression Analysis Results

This section employs a regression model predicated on the results derived from both the external and internal GDM applications of the TOPSIS and VIKOR methods. With respect to a sense of trust in the output, the results are meticulously compared to ascertain the most effective CP transfusion model out of the four examined methods. Table 7 reveals that the most successful results were achieved via the external GDM AHP-VIKOR model. This model demonstrated the highest R2 value at 0.971319 and the lowest scores for MSE (0.001046), RMSE (0.032348), and MAE (0.025314). In turn, demonstrating the model’s efficacy, it also shows a commendable R2 value of 0.969948 and the lowest respective values for MSE (0.000971), RMSE (0.031156), and MAE (0.024869). Consequently, the data confirm that the external GDM AHP-VIKOR model is the most effective approach used in this specific context. The best ranking results are presented in Table 8 according to the best model obtained.

These performance metrics collectively demonstrate that the external GDM AHP-VIKOR configuration provides the most stable prioritization behavior across independent samples. In particular, the minimal MSE and RMSE values reflect a narrow deviation between predicted and actual prioritization outputs, while an R^2^ exceeding 0.97 indicates strong explanatory power. Such results confirm the reliability of the ranking mechanism and reinforce its suitability for deployment in clinical or telemedicine settings where decision accuracy is essential.

Both sets of patient-donor data provide clear ordinal rankings of performance metrics, showing that the external GDM AHP-VIKOR model is, by far, the most robust, as demonstrated by the highest R^2^ values and lowest error scores. This suggests that VIKOR excels in decision environments with conflicting criteria. However, this study goes beyond existing work by validating the model statistically and integrating it with ABO-based matching logic. The high predictive power confirms the potential for real-time implementation in telemedicine scenarios, where decision latency and accuracy are critical.

## 5. Correlation-Based Approaches to Establish Trustworthiness

The trustworthiness of the study analysis hinges upon the use of robust correlation methods to compute Spearman’s correlation coefficient (rs) scores, as presented in Equation (22). Using these powerful techniques, we explore the relationships among all pairs of biomarker criteria (C 1 = PAO2/FIO, C 2 = CRB, C 3 = IL-6, C4 = Albumin, and C5 = IgM) present in the viral disease patient dataset with a ranking value (scoring). Skilled in detecting monotonic relationships, these techniques are innately suited for scrutinizing all criteria pairs. The aim of the presented study is twofold: to unveil marked linear relationships between all pairs of criteria and concurrently determine and assign the strength and direction of these relationships via the rs coefficient [70].

(22) $$r_{s}=1-\frac{6\sum \;D^{2}}{n\left(n^{2}-1\right)}$$

where D refers to the difference between the two ranks of each observation and n represents the number of observations. This equation illustrates the rs correlation coefficient calculation process, which is able to measure both the strength and direction of the monotonic relationship between the subsequently connected criteria. As shown in Table 9 and Table 10, the reliable/rigorous computation of the rs correlation has been demonstrated with r values assigned to each pair of criteria for both patients/donors, confirming the trustworthiness of the correlations established.

According to the findings presented in Table 9, the internal GDM AHP-TOPSIS demonstrates that ‘C1’ has the strongest positive association, with a ‘Scoring’ value of 0.569. On the other hand, the variable labeled ‘C3’ has the most significant negative correlation with the variable ‘Scoring’, as indicated by a correlation coefficient of −0.557. According to the external GDM AHP-TOPSIS analysis, the variable ‘C1’ is most positively correlated with the ‘Scoring’ variable, with a correlation coefficient of 0.648. On the other hand, the variable ‘C3’ has the largest negative connection, with a correlation coefficient of −0.495. The internal GDM AHP-VIKOR analysis reveals that ‘C4’ has the highest positive correlation with ‘C5’, with a correlation coefficient of 0.144. Additionally, ‘C3’ has the most substantial negative correlation with ‘Scoring’, with a correlation coefficient of −0.707. Finally, the external GDM with the AHP-VIKOR method demonstrated that ‘C4’ presented the most significant positive correlation with ‘C5’, with a coefficient of 0.144. On the other hand, ‘C1’ has the greatest negative correlation with ‘Scoring’, with a coefficient of −0.661. According to Table 10, the variable ‘C5’ has the most positive correlation with the variable ‘Scoring’ in the internal GDM AHP-TOPSIS approach, with a correlation coefficient of 0.481. In contrast, the variable ‘C3’ has the most pronounced negative correlation in the domain of ‘Scoring’, with a coefficient of (−0.646). According to the external GDM AHP-TOPSIS model, the variable ‘C5’ exhibits the most positive correlation with the ‘Scoring’ values, with a correlation coefficient of 0.457. Conversely, the variable ‘C3’ has the most significant negative correlation with the ‘Scoring’ values, with a correlation coefficient of −0.635. The internal GDM AHP-VIKOR analysis reveals that variables ‘C1’ and ‘C2’ exhibit the most positive connection, with a correlation coefficient of 0.143. Additionally, the variable ‘C1’ has the most significant negative correlation with the ‘Scoring’ variable, with a correlation coefficient of −0.597. The external GDM AHP-VIKOR shows that ‘C1’ is the most positively correlated with the ‘C2’ value of 0.143, and ‘C1’ has the highest negative correlation, with a score of −0.658.

The proposed framework aligns with core principles of trustworthy clinical AI. Transparency is ensured through the interpretable AHP weighting process and the stepwise computations of TOPSIS and VIKOR. Accountability is embedded in the expert-in-the-loop design, with future deployment incorporating clinician override and audit-log mechanisms. Although demographic attributes are not available for formal fairness assessment, the biomarker-driven logic is consistent and can support equity auditing when expanded to multi-center datasets. These elements position the system within modern governance standards for safe and reliable AI-assisted decision-making [71].

## 6. State of the Art: Comparative Analysis

This section presents a benchmark tool in the form of a checklist to compare the proposed trusted integrated intelligent framework with existing methods in the literature. This popular approach allows a systematic assessment of the originality and technical depth of the framework by evaluating it across important functional dimensions drawn from the previous literature. With this in mind, the comparison focuses on five key points that delineate methodological and operational progress in the field. Comparative outcomes across these points and benchmark studies are shown in Table 11 [14,24,29,30,31].

**1st Point:** Development of a Decision-Support Method for Multi-Criteria Selection. The present study assesses whether each study systematically derives a formal, multicriteria decision process for integrating disparate clinical indicators. Previous frameworks developed by Klassen et al. [29] and Manupati et al. [30] selection procedures were limited or heuristic, whereas Kajova et al. [31] and Mohammed et al. [14] focused on specific biological parameters and not on the trade-offs between parameters. Park et al. [24] used only thresholds of antibodies; this was a single-criterion study. The proposed framework bridges this gap by quantifying the importance and trade-offs (conflicts) of five biomarkers. By means of analytic weighting, it equilibrates conflicting indicators with respect to type (benefit or cost) and, through this, delivers a transparently replicable prioritization model.

**2nd Point:** Incorporation of the Telemedicine Environment. The integration of telemedicine across the literature remains fragmented and conceptual. Manupati et al. [30] and Mohammed et al. introduced networked hospital coordination in [14] but did not support a real-time or interoperable framework. It embeds telemedicine at its heart: in a multi-institutional system in which experts can participate remotely, data can be exchanged in a distributed format, and patient-donor allocation can be automated into an executable workflow. The architecture transforms the framework into an on-demand, telemedicine-ready, decision-support platform, uniting clinical intelligence and geographical flexibility.

**3rd Point:** Integration of Regression-Based Validation Toward Trustworthy AI. Most benchmark studies either rely on descriptive statistics or lack empirical validation mechanisms. Only Park et al. [24] applied regression and receiver operating characteristic (ROC) analyses to associate antibody levels with patient outcomes, yet they did not perform cross-validation or performance generalization. The proposed framework introduces a quantitative reliability layer via regression-based validation with precise metrics (R^2^ = 0.971 319, MSE = 0.001 046, RMSE = 0.032 348, MAE = 0.025 314). Cross-validation confirms its stability and predictive consistency, satisfying the emerging principles of trustworthy, explainable, and empirically verified AI in healthcare.

**4th Point:** Implementation of an Intelligent Matching Process Compatibility-based donor/patient matching has been recognized in prior works but is seldom automated. Kajova et al. [31] ensured ABO/HLA safety through laboratory screening; Mohammed et al. [14] proposed rule-based matching; and Park et al. [24] examined nationwide donor pools without automated pairing. The proposed framework embeds ABO compatibility constraints directly into the allocation algorithm, ensuring safety and fairness while supporting dynamic multihospital scalability. This intelligent integration converts matching from a manual procedure into a data-driven optimization task that ensures equitable transfusion outcomes.

**5th Point:** Empirical Trust Verification and Correlation Analysis. Moreover, most previous studies on trust have bypassed validation with quantification. Few correlation analyses were registered in Kajova et al. [31] and Park et al. [24], but with no explicit reliability interpretation. This work outlines a framework that incorporates an empirical layer of trust verification: through correlation analyses, it evaluates whether the observed rankings of biomarkers are consistent with stable outcomes to assess their internal coherence. We transform trust into a quantifiable property, which strengthens the accountability and explainability of automated quality assurance systems.

## 7. Conclusions

This study introduced an intelligent and reliable concept of CP transfusion management for patients with viral infection by establishing a combined prioritization/whole blood donor verification/matching system. Through the integration of expert-driven reasoning and data-driven validation, the framework solves two long-standing obstacles to clinical AI: methodological fragmentation and the absence of quantified trust. The results show that the integration of multifactor decision logic accompanied by empirical performance verification makes CP allocation more transparent, accurate and reliable. A dataset of 80 patients and 80 healthy donors for five biomarkers produced highly consistent ranking results with an external-based group AHP-VIKOR model (R^2^ = 0.971), resulting in minimal error-measure values (MSE = 0.0010; RMSE = 0.032; MAE = 0.025). This finding indicates that the designed system is capable of identifying the optimal CP donors under ABO compatibility constraints for the most severely ill patients, with a high confidence level (90%). The correlation analysis also confirmed the internal consistency and validity of the model, demonstrating its greater applicability in real-time telemedicine. Beyond purely technical performance, this research provides, within a single decision-support pipeline, concrete means to make the AI engineer’s design objectives related to transparency, explainability and validation actionable, contributing to the general topic of trustworthy AI in healthcare. A principal limitation of this work is the modest sample size (80 patients and 80 donors), inherited from the source clinical study. Additionally, the dataset used in this study does not include established clinical severity indexes (e.g., SOFA or APACHE), which limits our ability to correlate the final priority scores with these independent severity measures. Although adequate for demonstrating methodological feasibility, this cohort constrains generalizability and increases the risk of model overfitting. The use of simple regression structures and external validation reduces this risk. Validation on larger, multi-center datasets will be essential to confirm robustness and strengthen the translational reliability of the proposed framework. Additionally, while the use of external validation and regression-based performance comparison provides an empirical robustness check, the framework does not yet evaluate ranking stability underweight perturbation. Future work will incorporate systematic ±5–20% weight variation alongside Spearman and Kendall τ correlations to quantify the resilience of the prioritization outcomes to uncertainty in expert-assigned criteria weights.

The proposed framework can serve as a foundation for future adaptive transfusion systems and other critical resource-allocation models where fairness and accountability are essential. Future work will extend this approach to dynamic, multidisease datasets and integrate explainable AI components and mobile telehealth interfaces to increase accessibility and clinician trust.

## Figures and Tables

**Table 1:** Patient-Donor Samples from the Dataset.

Patients	Biomarker Measurements				
	PAO2/FIO2	CRP	IL-6	Albumin	IgM ELISA
P1_A	128	93	244	21	94.09
P1_B	136	83.64	168	24	154
P1_O	182	141.78	78	31	263
**Donors**	**Biomarker Measurements**				
D1_A	453	1.3	1.4	41.6	64.99
D1_B	425	3.96	1.98	44.44	32.01
D1_AB	449	2.97	4.95	47.47	37.83
D1_O	445	5.94	3.96	55.55	35.89

PAO2/FIO2 > 300, CRP, mg/L (<8), IL-6, pg/mL (Cytokines; normal range, 0–7), Albumin (40–55) g/L, IgM ELISA titer (<200), P = Patient, D = Donor.

**Table 2:** Prioritization DM for patients-donors.

Criteria/Patients	C1	C2	C3	C4	C5
Patient 1	C1-P1	C2-P1	C3-P1	C4-P1	C5-P1
Patient 2	C1-P2	C2-P2	C3-P2	C4-P2	C5-P2
Patient 3	C1-P3	C2-P3	C3-P3	C4-P3	C5-P3
Patient *n*	C1-P80	C2-P80	C3-P80	C4-P80	C5-P80
**Criteria/Donors**	**C1**	**C2**	**C3**	**C4**	**C5**
Donor 1	C1-D1	C2-D1	C3-D1	C4-D1	C5-D1
Donor 2	C1-D2	C2-D2	C3-D2	C4-D2	C5-D2
Donor 3	C1-D3	C2-D3	C3-D3	C4-D3	C5-D3
Donor *n*	C1-D80	C2-D80	C3-D80	C4-D80	C5-D80

C1= PAO2/FIO2 > 300, C2 = CRP, mg/L (<8), C3 = IL-6, pg/mL (Cytokines; normal range, 0–7), C4 = Albumin (40–55) g/L, C5 = IgM ELISA titer (<200), P = Patient, D = Donor.

**Table 3:** Nine scales of pairwise comparisons.

Intensity of Importance	Definition	Explanation
1	Equal importance	Two activities contribute equally to the objective
3	Weak importance of one over another	Experience and judgment slightly favor one activity over another
5	Essential or strong importance	Experience and judgment strongly favor one activity over another
7	Demonstrated importance	Activity is strongly favored and its dominance is demonstrated in practice
9	Absolute importance	The evidence favoring one activity over another is of the highest possible order of affirmation
2, 4, 6, 8	Intermediate values between the two adjacent judgments	When compromise is needed

**Table 4:** Weights assigned to biomarker criteria by the three experts via the AHP.

Biomarker Criteria/Experts	C1	C2	C3	C4	C5
First Expert Weights	0.343	0.067	0.407	0.086	0.098
Second Expert Weights	0.054	0.118	0.283	0.054	0.491
Third Expert Weights	0.427	0.199	0.199	0.061	0.113

**Table 5:** First ten patients-donors ranked via external/internal GDM for TOPSIS and VIKOR.

	Rank	External GDM				Internal GDM				
		Patients	Score	Donors	Score	Patients	Score	Donors	Score	Patients
TOPSIS	1	P3_AB	0.7471	D20_AB	0.6801	P3_AB	0.7429	D20_AB	0.6924	P3_AB
	2	P4_O	0.7289	D8_A	0.61142	P4_O	0.7258	D8_A	0.6472	P4_O
	3	P14_O	0.7215	D17_A	0.58684	P14_O	0.7099	D17_A	0.6037	P14_O
	4	P7_O	0.6854	D1_A	0.57641	P20_AB	0.6936	D1_A	0.5671	P20_AB
	5	P19_AB	0.6832	D6_O	0.53056	P7_O	0.6836	D6_O	0.5605	P7_O
	6	P16_A	0.6810	D6_A	0.52952	P16_A	0.6750	D14_A	0.5466	P16_A
	7	P20_AB	0.6749	D14_A	0.50619	P1_O	0.6671	D6_A	0.5397	P1_O
	8	P1_O	0.6600	D18_B	0.49735	P19_AB	0.6647	D18_B	0.5267	P19_AB
	9	P8_AB	0.6465	D11_A	0.48283	P8_AB	0.6644	D11_A	0.5230	P8_AB
	10	P17_O	0.5996	D7_A	0.47616	P12_A	0.5743	D7_A	0.4418	P12_A
VIKOR	1	P8_A	0.0580	D11_O	0	P8_A	0.002	D11_O	0.000	P8_A
	2	P16_O	0.0853	D20_O	0.1663	P16_O	0.014	D20_O	0.180	P16_O
	3	P20_A	0.1703	D2_AB	0.2485	P20_A	0.106	D2_AB	0.230	P20_A
	4	P15_A	0.1844	D19_B	0.2574	P15_A	0.124	D4_O	0.300	P15_A
	5	P20_O	0.2004	D17_O	0.2655	P17_B	0.178	D19_B	0.310	P17_B
	6	P17_B	0.2076	D4_O	0.2794	P20_O	0.185	D17_O	0.326	P20_O
	7	P6_O	0.2123	D18_AB	0.2908	P5_A	0.212	D18_AB	0.327	P5_A
	8	P5_A	0.2652	D10_AB	0.2967	P6_O	0.218	D10_AB	0.328	P6_O
	9	P4_B	0.2735	D3_AB	0.3019	P13_B	0.276	D3_AB	0.339	P13_B
	10	P6_AB	0.2799	D7_O	0.3221	P5_O	0.283	D6_AB	0.341	P5_O

**Table 6:** Matching results for external GDM AHP-TOPSIS and GDM AHP-VIKOR.

Rank	GDM AHP-TOPSIS		GDM AHP-VIKOR	
	Patients	Suitable Donors	Patients	Suitable Donors
1	P3_AB	D13_AB	P8_A	D11_A
2	P4_O	D14_O	P16_O	D6_O
3	P14_O	D5_O	P20_A	D14_A
4	P7_O	D19_O	P15_A	D8_A
5	P19_AB	D8_AB	P20_O	D5_O
6	P16_A	D19_A	P17_B	D18_B
7	P20_AB	D5_AB	P6_O	D3_O
8	P1_O	D13_O	P5_A	D3_A
9	P8_AB	D4_AB	P4_B	D8_B
10	P17_O	D12_O	P6_AB	D8_AB

**Table 7:** Regression analysis results for patients/donors.

Alternatives	MODEL	MSE	RMSE	MAE	R2
Patients	Internal GDM AHP-TOPSIS	0.002213	0.047038	0.039334	0.865505
	External GDM AHP-TOPSIS	0.001668	0.040841	0.033125	0.903908
	Internal GDM AHP-VIKOR	0.006353	0.079706	0.061567	0.877693
	External GDM AHP-VIKOR	0.001046	0.032348	0.025314	0.971319
Donors	Internal GDM AHP-TOPSIS	0.005802	0.076168	0.058001	0.630192
	External GDM AHP-TOPSIS	0.004773	0.06909	0.052701	0.672134
	Internal GDM AHP-VIKOR	0.003928	0.062674	0.049589	0.891921
	External GDM AHP-VIKOR	0.000971	0.031156	0.024869	0.969948

**Table 8:** Best selected model ranking results for patients/donors.

Rank	Patients	Final Rank	Donors	Final Rank
1	P8_A	0.058065821	D1_A	0.539869733
2	P16_O	0.085300918	D2_A	0.430855744
3	P20_A	0.170316404	D3_A	0.788494984
4	P15_A	0.184420827	D4_A	0.622832314
5	P20_O	0.200438641	D5_A	0.594066766
6	P17_B	0.207678132	D6_A	0.666066921
7	P6_O	0.212389723	D7_A	0.395474276
8	P5_A	0.265279477	D8_A	0.825240058
9	P4_B	0.273568837	D9_A	0.387741421
10	P6_AB	0.279913825	D10_A	0.726689541

**Table 9:** Correlation results for the criteria and scoring for patients.

Model 1			Model 2			Model 3			Model 4		
V1	V2	r Value	V1	V2	r Value	V1	V2	r Value	V1	V2	r Value
C1	Scoring	0.569	C1	Scoring	0.648	C3	Scoring	−0.707	C1	Scoring	−0.661
C3	Scoring	−0.557	C3	Scoring	−0.495	C1	Scoring	−0.527	C3	Scoring	−0.57
C5	Scoring	0.388	C5	Scoring	0.383	C5	Scoring	−0.357	C5	Scoring	−0.455
C2	C4	−0.217	C2	C4	−0.217	C2	C4	−0.217	C2	Scoring	−0.238
C4	C5	0.144	C4	C5	0.144	C2	Scoring	−0.18	C2	C4	−0.217
C2	C5	0.133	C2	C5	0.133	C4	C5	0.144	C4	C5	0.144
C1	C4	−0.096	C1	C4	−0.096	C2	C5	0.133	C2	C5	0.133
C3	C5	0.089	C3	C5	0.089	C4	Scoring	0.096	C1	C4	−0.096
C4	Scoring	0.06	C4	Scoring	0.077	C1	C4	−0.096	C3	C5	0.089
C3	C4	−0.036	C2	Scoring	−0.055	C3	C5	0.089	C4	Scoring	0.039
C2	Scoring	−0.02	C3	C4	−0.036	C3	C4	−0.036	C3	C4	−0.036
C1	C3	0.017	C1	C3	0.017	C1	C3	0.017	C1	C3	0.017
C2	C3	−0.012	C2	C3	−0.012	C2	C3	−0.012	C2	C3	−0.012
C1	C5	−0.011	C1	C5	−0.011	C1	C5	−0.011	C1	C5	−0.011
C1	C2	0.01	C1	C2	0.01	C1	C2	0.01	C1	C2	0.01

**Table 10:** Correlation results for criteria and scoring for donors.

Model 1			Model 2			Model 3			Model 4		
V1	V2	r value	V1	V2	r value	V1	V2	r value	V1	V2	r value
C3	Scoring	−0.646	C3	Scoring	−0.635	C1	Scoring	−0.597	C1	Scoring	−0.658
C5	Scoring	0.481	C5	Scoring	0.457	C3	Scoring	−0.57	C3	Scoring	−0.495
C2	Scoring	−0.3	C1	Scoring	0.33	C2	Scoring	−0.326	C5	Scoring	−0.426
C1	Scoring	0.257	C2	Scoring	−0.284	C5	Scoring	−0.312	C2	Scoring	−0.245
C2	C5	−0.177	C2	C5	−0.177	C2	C5	−0.177	C2	C5	−0.177
C1	C2	0.143	C1	C2	0.143	C1	C2	0.143	C1	C2	0.143
C4	Scoring	0.139	C1	C4	0.132	C1	C4	0.132	C1	C4	0.132
C1	C4	0.132	C4	Scoring	0.127	C1	C3	−0.103	C1	C3	−0.103
C1	C3	−0.103	C1	C3	−0.103	C3	C4	−0.097	C3	C4	−0.097
C3	C4	−0.097	C3	C4	−0.097	C3	C5	0.079	C3	C5	0.079
C3	C5	0.079	C3	C5	0.079	C2	C4	0.074	C2	C4	0.074
C2	C4	0.074	C2	C4	0.074	C2	C3	0.056	C4	Scoring	−0.057
C2	C3	0.056	C2	C3	0.056	C4	C5	−0.03	C2	C3	0.056
C4	C5	−0.03	C4	C5	−0.03	C4	Scoring	−0.028	C4	C5	−0.03
C1	C5	0.023	C1	C5	0.023	C1	C5	0.023	C1	C5	0.023

**Table 11:** Comparison points in the benchmarks and proposed framework.

Comparison Points/Benchmarks		Benchmark#1 [29]	Benchmark#2 [30]	Benchmark#3 [31]	Benchmark#4 [14]	Benchmark#5 [24]	Proposed Framework
1st Development of a Decision-Support Method for Multi-Criteria Selection.	(a) Criteria Importance	ꭓ	ꭓ	✔	✔	✔	✔
	(b) Criteria Trade-offs	ꭓ	ꭓ	ꭓ	✔	ꭓ	✔
	(c) Criteria Conflicts	ꭓ	ꭓ	✔	✔	ꭓ	✔
2nd Incorporation of the Telemedicine Environment.		ꭓ	✔	ꭓ	✔	✔	✔
3rd Integration of Regression-Based Validation Toward Trustworthy AI.	(a) Use of quantitative performance indices	✔	✔	✔	ꭓ	✔	✔
	(b) Application of cross-validation to demonstrate generalizability	ꭓ	ꭓ	ꭓ	ꭓ	ꭓ	✔
4th Implementation of an Intelligent Matching Process.	(a) Compatibility safety embedded	✔	✔	✔	✔	✔	✔
	(b) Demonstration of scalability to multihospital datasets	ꭓ	✔	ꭓ	ꭓ	✔	✔
5th Empirical Trust Verification and Correlation Analysis.		ꭓ	ꭓ	✔	ꭓ	✔	✔
Total		33.3%	44.4%	55.5%	66.6%	44.4%	100%

Note: ꭓ means no, ✔ means yes.

## Data Availability

The data of the paper, which support the analysis and results of this paper, are available with the corresponding author and the data can be obtained from the authors upon request.

## Abbreviations

The following abbreviations are used in this manuscript:

CP	Convalescent plasma
MCDM	Multicriteria decision-making
AHP	Analytic hierarchy process
TOPSIS	Order preference by similarity to ideal solution
VIKOR	Višekriterijumsko kompromisno rangiranje
GDM	Group decision-making
